# Capillary microscopy is a potential screening method for connective tissue disease in children with Raynaud’s phenomenon

**DOI:** 10.1186/s12969-022-00671-0

**Published:** 2022-02-08

**Authors:** Claudette A. Farenhorst, Anniek M. Roon, Anne I. Gessel, Alja J. Stel, Hendrika Bootsma, Wineke Armbrust, Douwe J. Mulder

**Affiliations:** 1 Department of Rheumatology and Clinical Immunology, University of Groningen, University Medical Centre Groningen, Groningen, The Netherlands; 2 Deptartment of Internal Medicine, division Vascular Medicine, University of Groningen, University Medical Centre Groningen, Groningen, the Netherlands; 3 Department of Pediatric Rheumatology and Immunology, Beatrix Children’s Hospital, University of Groningen, University Medical Centre Groningen, Groningen, The Netherlands

**Keywords:** Raynaud’s phenomenon, Nailfold capillary microscopy, Connective tissue disease

## Abstract

**Background:**

Nailfold capillary microscopy (NCM) is a cornerstone in the diagnosis of Systemic Sclerosis (SSc) in adulthood. Although Raynaud’s phenomenon (RP) is very common in childhood, studies on diagnostic methods to differentiate between primary RP (PRP) and secondary RP (SRP) at a young age are scarce. The aim of this study was to determine the value of NCM in differentiating between PRP and SRP in children and adolescents with RP.

**Methods:**

In this nested case–control study, 83 patients diagnosed with RP and having underwent NCM in childhood were retrospectively included. Based on whether they were diagnosed with a connective tissue disease (CTD) during follow-up, patients were classified as PRP or SRP. NCM was performed by a vascular technician. PRP and SRP patients were compared on demographics, NCM and serology. Variables associated with SRP were included in a multivariate logistic regression model. Predictive values were calculated for NCM, ANA positivity and the combination of NCM and ANA positivity.

**Results:**

At the time of the NCM, the mean age of the RP patients was 15.4 ± 2.3 years. Of these patients, 78.3% were classified as PRP and 21.7% as SRP at mean follow-up of 6.4 ± 3.20 years. CTDs were miscellaneous, with only one patient having developed SSc. Of the NCM parameters, only capillary loss was associated with SRP (*p* = 0.01). In a multivariate logistic regression model including ANA, capillary loss was not a predictor of SRP. In a model without ANAs, capillary loss was an independent predictor (OR = 3.98, CI 95% 1.22–12.99). Capillary loss had a sensitivity of 44.4% and a specificity of 84.4% for SRP. ANA combined with capillary loss had a sensitivity of 66.7% and a specificity of 85.7%.

**Conclusion:**

Whereas RP in adulthood is most strongly associated with SSc, children with RP seem to be at risk for developing other CTDs with less apparent NCM abnormalities. Of all NCM findings, only capillary loss was predictive for SRP. NCM did not add to the predictive value of ANA screening. However, with a specificity of 84.4% and being non-invasive, NCM shows potential as a screening method for SRP. More research with a larger study population is required before drawing conclusions.

## Introduction

Nailfold capillary microscopy (NCM) is a cornerstone in the diagnosis of systemic sclerosis (SSc) in adulthood [1]. Although Raynaud’s phenomenon (RP) is common in childhood with a prevalence of 14.9% in children aged 12–15 years [2–4], studies on diagnostic methods to differentiate between primary RP (PRP) and secondary RP (SRP) at a young age are scarce. In PRP no cause can be identified, whereas SRP occurs as part of an underlying disease, most commonly a connective tissue disease (CTD) [5]. The disease course of CTDs varies, and is often associated with severe morbidity and sometimes mortality. In children and adolescents, early diagnosing and optimal treatment at an early stage of the disease can improve prognosis significantly [6]. Therefore, it is important to distinguish between PRP and SRP early.

NCM is a non-invasive method for visualizing the microcirculation of the nailbed. In adults, NCM has been established as an effective method for predicting the development of SSc in RP patients, especially when combined with serology [1]. Capillaroscopic findings that should alert the physician to the possibility of undetected SSc are hemorrhages, loss of capillaries, widened capillaries and giant capillaries [7, 8]. These capillaroscopic abnormalities are mainly seen in SSc, but have been reported in other CTDs as well [9, 10]. For rheumatoid arthritis (RA), it was reported that 20.9% of the patients showed capillaroscopic abnormalities [11]. There are few studies on capillaroscopic abnormalities in Sjögren’s syndrome (SS) and systemic lupus erythematosus (SLE). However, the studies that have been performed found significantly more capillaroscopic abnormalities in SS and SLE patients compared to the healthy population [12, 13].

For children with RP, however, guidelines for initial investigation and subsequent follow-up are based on limited evidence [14, 15]. Only one pediatric prospective study has been conducted previously, and found results similar to studies in adults with RP. In this study, SSc-related capillary changes significantly correlated with development of SSc-spectrum disorders in the near future. Future development of other CTDs could not be predicted by specific capillary changes [15, 16].

A screening method for CTDs that is often performed in children is serological testing for ANAs. A high ANA titer is suggestive of a CTD. Antibodies against one or more extractable nuclear antigens (ENAs) suggest a specific CTD such as SSc, SS or SLE [17]. ANA and anti-ENA antibodies are important diagnostic markers of systemic rheumatic diseases, especially because for many diseases they are part of the diagnostic criteria.

The general aim of this study was to determine the value of NCM in differentiating between PRP and SRP in children and adolescents with RP.

## Methods

### Patient selection

Patients were retrospectively included in this case–control study. Inclusion criteria were an age ≤ 18 years, having established RP, having underwent an NCM at the vascular laboratory of the University Medical Center Groningen between September 2008 and March 2019 and having accessible capillaries at the time of the NCM. All included participants provided informed consent. RP was defined as a history of ≥ two phases of color change (white, blue, red) with/without discomfort of the hands, induced by exposure to cold temperature. Patients were considered to have SRP if they were diagnosed with a CTD based on current classification criteria or had incomplete disease but were deemed SRP by their treating physician [18–23]. Adults criteria were used for this, as all patients were adults at the time of follow-up. The used criteria were the 2013 American College of Rheumatology (ACR)/European League Against Rheumatism (EULAR) criteria for SSc [18], the 2019 ACR/EULAR criteria for SLE [19], the 2017 EULAR/ACR criteria for adult and juvenile idiopathic inflammatory myopathies [20], the 2010 ACR/EULAR criteria for RA [21], and the 2016 ACR/EULAR criteria for SS [22]. For incomplete disease, the following criteria were used: LeRoy criteria for incomplete SSc (iSSc) [23], fulfilling 3 or 4 ACR criteria for SLE for incomplete SLE (iSLE) [19], fulfilling 3 ACR criteria for SS for incomplete SS (iSS) [22] and criteria proposed by Mosca et al. for undifferentiated CTD (UCTD) [24]. Patients were diagnosed with mixed CTD (MCTD) using the criteria defined by Kasukawa et al. [25]. Whether and when patients received this diagnosis, was retrieved from the electronic patient record. Two investigators adjudicated independently and a consensus was reached in all cases.

### Nailfold capillaroscopy

NCM assessments were performed once in all patients. For all NCM assessments, immersion oil was applied on the nailfold to increase transparency. In addition, NCM was performed after an adaptation period of ≥ 15 min at room temperature (23 °C). The third and fourth digits of both hands were assessed. The vascular technicians all followed the same protocol to minimize differences between the technicians. All NCM assessments were supervised by a medical specialist. For uncertain cases, a consensus based conclusion was reached between the vascular technician and the medical specialist [26].

Up until January 2014, 55 NCM were performed with an Olympus BHMJ FW-32362 (Tokyo, Japan), with a Grundig FA-85 Z/W video camera (Fürth/Bay, Germany) and an Osram XBO 75 W xenon lamp (Berlin, Germany) with a 180 × enlargement. The images were assessed by one of five vascular technicians. The presence of dilated capillaries, giant capillaries and hemorrhages were judged visually over the whole distal row of the nailfold. The number of capillaries were counted in a 3 mm grid [26].

After January 2014, 28 NCM were performed with an Olympus BXFM (Olympus, Tokyo, Japan), with Olympus CellSens software (Olympus, Tokyo, Japan) with a 180 × enlargement. A 3 mm grid of the distal row of the capillaries was judged visually by one of two vascular technicians. In a 3 mm grid the capillary density per 3 mm of the distal row of the nailfold and haemorrhages was counted, the apex was measured to determine giant capillaries (> 50 µm) and dilated capillaries (> 20 µm, < 50 µm).

Capillary loss was defined as < 6 capillaries per mm of the distal row of the nailfold per finger. Patterns were classified according to the criteria defined by Cutolo et al. [27]. A normal pattern was indicated by no capillary loss, a mean number of dilated capillaries of ≤ 3 and no giant capillaries; a nonspecific pattern was indicated by a number of > 3 dilated capillaries and/or capillary loss, in the absence of giant capillaries; an early SSc pattern was indicated by ≥ 1 giant capillaries without loss of capillaries or hemorrhages; an active SSc pattern was indicated by ≥ 1 giant capillaries combined with capillary loss and/or hemorrhages; and a late SSc pattern was indicated by severe loss of capillaries with none or few giant capillaries, none or few hemorrhages and signs of neovascularization [27, 28].

### Laboratory assessments

ANAs were tested by indirect immunofluorescence. When present, they were classified as speckled, homogeneous, anticentromere, or nucleolar. ANAs were defined positive for titer ≥ 1:80. Antibodies against ENAs were measured by fluorescent enzyme immunoassay. Anti-ENA antibodies were defined as positive for titer > 10 U/ml. With this cut-off value, the chance of having false positives is low. Autoantibodies analyzed in this screening were those against U1-RNP, RAP-70, Sm, SSA, SSB, Jo1, topoisomerase-1(scl-70) and CENP-B [28].

### Statistical analyses

Statistical analyses were performed using IBM SPSS statistics (version 23).

An unpaired t-test, Mann–Whitney U test, Chi-Square test or Fisher’s Exact test was used to compare the baseline characteristics of patients with PRP and SRP. The variables associated with SRP (*p* < 0.1) in the univariate analysis were entered into a multivariate logistic regression model. The type of RP (PRP versus SRP) was established as the dependent variable of the model. Statistical significance was achieved when *p* < 0.05. Two multivariate logistic regression models were performed using the Enter method; one with and one without ANA positivity included. These models were corrected for age. Sensitivity and specificity were calculated for regression models with only ANAs, only capillary loss and ANAs together with capillary loss. Sensitivity and specificity of different cut off points for numbers of capillaries were determined with ROC curve analysis. Lastly, a multivariate logistic model with both ANA and anti-ENA screening was performed in which an interaction variable was created of ANA and anti-ENA.

## Results

### Patient characteristics

A number of 98 patients with an age of ≤ 18 years underwent NCM between 2008 and 2019. Of these patients, 14 were excluded because RP was not established based on the physicians judgement and one was excluded because the capillaries were not assessable. The remaining 83 patients were included in this study. Patient characteristics are shown in Table 1. The age at which the NCM was conducted ranged from 7.3 to 18.0 years, with a mean age of 15.4 ± 2.26 years. The male to female ratio was approximately 1:3.5. After an average follow-up period of 6.4 ± 3.20 years, 65 (78.3%) of the patients were found to have PRP. SRP was established in 18 (21.7%) of the patients. At the time of follow-up, all patients were adults.

**Table 1:** Baseline characteristics for the whole study population, and the PRP group and SRP group separately

**Characteristics**	**All patients** ***n* = 83**	**PRP-patients** ***n* = 65**	**SRP-patients** ***n* = 18**	***P*-value**
**Demographics**				
Age at NCM, mean ± sd	15.4 ± 2.26	15.7 ± 1.81	14.3 ± 3.26	**0.10**
Female gender, n (%)	64 (77.1)	49 (75.4)	15 (83.3)	0.48
**NCM**				
Capillary loss (< 6/mm), n (%)^a^	18 (22.0)	10 (15.6)	8 (44.4)	**0.01**
Dilated capillaries (> 3/3 mm), n (%)	40 (48.2)	32 (49.2)	8 (44.4)	0.72
Giant capillaries (> 0/3 mm), n (%)	21 (25.3)	15 (23.1)	6 (33.3)	0.38
Haemorrhages (> 0/3 mm), n (%)	14 (16.9)	10 (15.4)	4 (22.2)	0.49
Number of capillaries/mm, mean ± sd^a^	6.9 ± 0.96	7.0 ± 0.8	6.4 ± 1.3	**0.06**
Number of dilated capillaries/3 mm, mean ± sd	4.1 ± 4.36	4.3 ± 4.27	3.8 ± 4.8	0.43
Number of giant capillaries/3 mm, mean ± sd	0.3 ± 0.93	0.2 ± 0.70	0.5 ± 1.42	0.39
Number of haemorrhages/3 mm, mean ± sd	0.2 ± 0.67	0.2 ± 0.65	0.3 ± 0.73	0.42
**Serology**				
Positive ANA screening, n (%)^a^	21 (25.6)	9 (14.1)	12 (66.7)	** < 0.001**
Positive anti-ENA screening, n (%)^b^	12 (14.8)	3 (4.8)	9 (50.0)	** < 0.001**

The last column shows *p*-value (in bold: *p* < 0.10) for comparison between PRP and SRP

*Abbreviations:* *ANA* Antinuclear Antibodies, *ENA* Extractable Nuclear Antigens, *NCM* Nailfold Capillary Microscopy, *PRP* Primary Raynaud’s Phenomenon, *SRP* Secondary Raynaud’s Phenomenon, *SSc* Systemic Sclerosis

^*a*^*1 missing value (1.5%). *^*b*^*2 missing values (2.4%)*

Of the patients with SRP, 11 (61.1%) had received a definite classifiable diagnosis, whereas 7 (38.9%) had an undifferentiated or early stage CTD. Of patients with SRP, 4 (22.2%) had MCTD, 4 (22.2%) had UCTD, 3 (16.7%) had SLE, 2 (11.1%) had dermatomyositis (DM), 1 (5.6%) had SSc, 1 (5.6%) had SS, 1 (5.6%) had iSLE, 1 (5.6%) had iSSc and 1 (5.6%) had iSS.

In 16 (88.9%) of the 18 SRP patients, RP was already present at first presentation. In 7 (38.9%) of these patients, it was the cardinal symptom. Other common first presenting symptoms were arthralgia and cutaneous symptoms. All patients that presented with RP as the cardinal symptom showed other symptoms at presentation as well, most commonly joint complaints.

### Univariate analysis

#### Demographics

Table 1 shows the baseline characteristics. The age at baseline showed a trend towards a younger age of SRP patients (*p* = 0.10). The male to female ratio was not different between PRP and SRP (*p* = 0.48).

#### Nailfold capillary microscopy

15 (23.1%) of the PRP patients showed an SSc-pattern on NCM, of which 6 (9.2%) showed early changes and 9 (13.8%) showed active changes. 6 (33.3%) of the SRP patients showed an SSc-pattern on NCM, all of which showed active changes. 22 (33.8%) of the PRP patients and 6 (33.3%) of the SRP patients showed non-specific changes. Analysis of the NCM data showed a trend that SRP patients had fewer capillaries compared to PRP patients, with a mean number of capillaries per mm of 6.4 ± 1.3 in the SRP group and 7.0 ± 0.8 in the PRP group (*p* = 0.06). In the SRP group, 44.4% of the patients had capillary loss, whereas in the PRP group, 15.6% of the patients had capillary loss (*p* = 0.01). No significant differences were found for the number of dilated capillaries, giant capillaries and haemorrhages when comparing PRP to SRP patients (Table 1).

#### Serology

ANA positivity was seen significantly more often present in the SRP group compared to the PRP group; 66.7% of the SRP patients and 14.1% of the PRP patients showed ANA positivity (*p* < 0.001). All PRP patients with a positive ANA had a titer of 1:160 or lower. Of the SRP patients with a positive ANA titer 7 (58%) had a titer of 1:640, 1 (8%) had a titer of 1:320, 3 (25%) had a titer of 1:160 and 1 (8%) had a titer of 1:80. In most patients a coarse speckled pattern was seen. Other patterns that were seen less frequently are fine speckled, nucleolar and homogeneous patterns. Anti-ENA antibodies were also seen significantly more often in the SRP group compared to the PRP group: 50.0% of the SRP patients and 4.8% of the PRP patients showed anti-ENA antibodies (*p* = 0.001) (Table 1). PRP patients had a mean anti-ENA titer of 1.37 ± 5.71 and SRP patients had a mean anti-ENA titer of 12.79 ± 14.99.

### Multivariate analysis

The results of the multivariate logistic regression analysis including all univariately significant variables are shown in Table 2. Children with a positive ANA screening were 11.19 times (OR 11.19; 95% CI 3.07–40.79) more likely to have SRP compared to children with a negative ANA screening. A younger age at baseline and less capillaries at NCM were both not predictive of SRP in this model. Table 3 shows the multivariate logistic regression analysis using the same variables with the exclusion of ANA positivity. According to this model, children with a younger age at baseline were less likely to have SRP in comparison to older children (OR 0.79; 95% CI 0.63–1.00). In addition, children with capillary loss were 3.98 times (OR 3.98; CI 1.22–12.99) more likely to have SRP in comparison to children without capillary loss. Predictive values of ANA screening, capillary loss on NCM and the combination of both are presented in Table 4. ANA positivity had a sensitivity of 66.7% and a specificity of 85.9%. Capillary loss had a sensitivity of 44.4% and a specificity of 84.4%. ANA positivity combined with capillary loss had a sensitivity of 66.7% and a specificity of 85.7%.

**Table 2:** Predictive value of baseline variables for SRP according to multivariate logistic regression model

**Independent variables**	**B(SE)**	**OR (95% CI)**	***P*-value**
Age at NCM	-0.25 (0.14)	0.78 (0.60–1.02)	0.065
Capillary loss (< 6/mm)	1.07 (0.71)	2.92 (0.73–11.63)	0.130
Positive ANA screening	2.41 (0.66)	11.13 (3.03–40.89)	** < 0.001**

Variables associated with SRP in the univariate analysis (*p* < 0.10) were considered for inclusion in the multivariate logistic regression model. Variables included in the model were: age at NCM, capillary loss and ANA screening. The number of capillaries was not included because of its correspondence to capillary loss. Chi2 (df = 3) = 24.42. Nagelkerke pseudo R2 = 0.40. % PRP correctly predicted = 92.1. % SRP correctly predicted = 38.2. % overall correctly predicted = 80.2. Negative B values are negatively correlated to SRP. The last column shows *p*-value (in bold: *p* < 0.05)

*Abbreviations:* *ANA* Antinuclear Antibodies, *CI* Confidence Interval, *NCM* Nailfold Capillary Microscopy, *OR* Odds Ratio, *SE* Standard Error

**Table 3:** Multivariate logistic regression model showing predictive value of baseline variables for SRP excluding ANA positivity

**Independent variables**	**B(SE)**	**OR (95% CI)**	***P*-value**
Age at NCM	-0.23 (0.12)	0.79 (0.63–1.00)	**0.050**
Capillary loss (< 6/mm)	1.38 (0.60)	3.98 (1.22–12.99)	**0.022**

Variables associated with SRP in the univariate analysis (*p* < 0.10) were considered for inclusion in the multivariate logistic regression model. Variables included in the model were: age at NCM and capillary loss. The number of capillaries was not included because of its correspondence to capillary loss. Chi2 (df = 3) = 9.94. Nagelkerke pseudo R2 = 0.18. % PRP correctly predicted = 98.4. % SRP correctly predicted = 27.8. % overall correctly predicted = 82.9. ANA positivity was not included in this model. Negative B values are negatively correlated to SRP. The last column shows *p*-value (in bold: *p* < 0.05).

*Abbreviations: CI* Confidence Interval, *NCM* Nailfold Capillary Microscopy, *OR* Odds Ratio, *SE* Standard Error

**Table 4:** Predictive value of logistic regression models comprising different independent variables for prediction of SRP

	**Sensitivity (%)**	**Specificity (%)**	**PPV (%)**	**NPV (%)**	**% correctly classified**
ANA screening	66.7	85.9	57.1	90.2	81.7
Capillary loss (< 6/mm)	44.4	84.4	44.4	84.4	75.6
ANA screening + Capillary loss (< 6/mm)	66.7	85.7	57.1	90.0	81.5

A classification cut-off of 0.44 was used for all models

*Abbreviations*: *ANA* Antinuclear Antibodies, *NPV* Negative Predictive Value, *PPV* Positive Predictive value

#### ANA and anti-ENA screening

We also ran a multivariate model with age and both ANA and anti-ENA screening. Because of the significant correlation between ANA and anti-ENA screening, an interaction variable ANA*anti-ENA was included in the model. The model showed that the interaction variable did not significantly contribute to the model (*p* = 0.647). ANA and anti-ENA screening were both independent predictors of SRP. ANA positivity (OR 6.41; 95% CI 1.17–35.02) had a slightly higher odds ratio for SRP than anti-ENA positivity (OR 5.74; 95% CI 1.02–32.32) (Table 5).

**Table 5:** Predictive value of ANA and anti-ENA screening for SRP according to multivariate logistic regression model

**Independent variables**	**B(SE)**	**OR (95% CI)**	***P*-value**
Age at NCM	-0.173 (0.15)	0.84 (0.62–1.14)	0.261
Positive ANA screening	1.86 (0.87)	6.41 (1.17–35.02)	**0.032**
Positive anti-ENA screening	1.75 (0.88)	5.74 (1.02–32.32)	**0.048**

Results were obtained with a multivariate linear regression model. Variables included in the model were: age at NCM, and ANA and anti-ENA screening. ANA*anti-ENA was included as an interaction variable (not shown in table). All variables were included using the Enter method. Chi2 (df = 3) = 18.59. Nagelkerke pseudo R2 = 0.40. % PRP correctly predicted = 94.4. % SRP correctly predicted = 55.6% overall correctly predicted = 81.5. Negative B values are negatively correlated to SRP. The last column shows the *p*-value (in bold: *p* < 0.05)

*Abbreviations*: *ANA* Antinuclear Antibodies, *ENA* Extractable Nuclear Antigens, *CI* Confidence Interval *NCM* Nailfold Capillary Microscopy, *OR* Odds Ratio, *SE* Standard Error

### ROC curve analysis

**Fig. 1:**
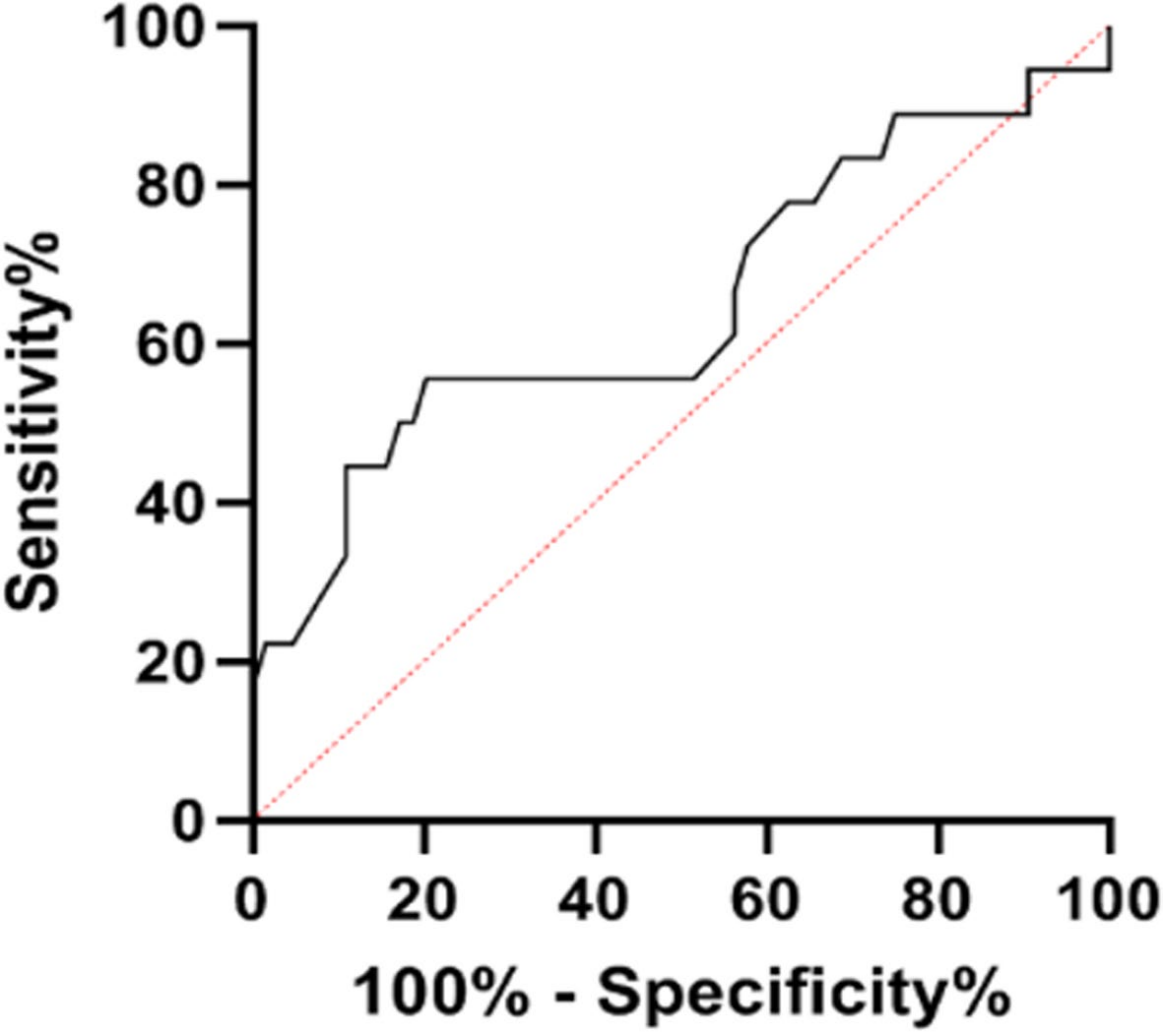
ROC curve of the number of capillaries as predictive variable for SRP

The ROC curve for the number of capillaries per mm is shown in Fig. 1. The results of the statistical analysis of the ROC curve are presented in Table 6. The ROC curve of the number of capillaries per mm had an area under the curve (AUC) of 0.65. However, this value was not significant (*p* = 0.06). Especially when numbers of capillaries were higher than approximately 6.7 per mm, they could not distinguish SRP from PRP. A cut-off value of 5.97 capillaries per mm (closest to the value used to define capillary loss) resulted in a sensitivity of 44.4% and a specificity of 84.4%.

**Table 6:** AUC in the ROC curve of the number of capillaries as predictive variable for SRP

**AUC**	**Std. Error**	**Asymptotic Sig**	**Asymptotic 95% CI**
0.648	0.08	0.06	0.485 – 0.811

*Abbreviations*: *AUC* Area Under the Curve, *CI* Confidence Interval

## Discussion

The aim of this study was to determine the value of NCM in differentiating between PRP and SRP in children and adolescents with RP. Capillary loss shown on NCM was associated with SRP. However, it did not add to the predictive value of ANAs. Other NCM characteristics were not associated with the presence of SRP.

This study showed that whereas SRP in adults is mostly associated with SSc, children and adolescents with SRP seem to mostly develop other CTDs. This is supported by the fact that only 1 of the 18 SRP patients developed SSc. However, 6 (33.3%) of the SRP had an SSc pattern with NCM, all of which showed active changes. Therefore, a study with more children should be performed to confirm whether children develop SSc less frequently than adults.

In this study, 22% of the patients were classified as SRP and 78% as PRP. At the time of referral, none of the patients were diagnosed with SRP yet. Some patients showed other symptoms at presentation, mostly joint complaints. For some patients, a CTD was suspected, but ANA and NCM were requested to further aid in diagnostics. In a few cases, patients showed ANA positivity as well as NCM abnormalities. These patients were still classified as PRP because they did not have symptoms suggestive of a CTD for longer than three years, which was one of the criteria for UCTD [24]. The mean time that passed between the start of RP and a definite diagnosis of a CTD was 2.1 years. These findings are comparable to those reported in the only other longitudinal study on childhood RP by Pavlov et al., which found SRP in 24% of the patients and PRP in 76% of the patients. The time between onset of RP and the diagnosis of a CTD was 2.4 years [15]. Both studies indicate that childhood RP is primary in most cases. However, in comparison to adult RP, childhood RP is more often secondary [29]. Other studies reported even higher proportions of SRP in children, ranging from 31 to 52% [15, 16, 30]. Pavlov et al. proposed as possible explanation the fact that the aforementioned studies only included children younger than 18 years, whereas their own study investigated children and adolescents younger than 20 years. However, our study only included patients below the age of 18 and also found a lower frequency of SRP. Therefore the cut-off point for age does not seem to be the cause of this difference. It is more likely that the difference in frequencies is partly attributable to differences in criteria chosen to establish RP and to classify CTDs.

The majority of children with SRP were diagnosed with MCTD, UCTD and SLE. SSc, DM and SS were seen less frequently. A possible explanation for this finding is that children with DM and SS are not always referred. Pavlov et al. reported a similar distribution of CTD diagnoses. Noteworthy is that Pavlov et al. found RA or juvenile idiopathic arthritis (JIA) in 4% of the patients and Nigrovic et al. found arthritis in 29% of the patients [15, 16]. Although the present study did find joint complaints to be a common early presenting symptom of juvenile CTDs, none of the children were diagnosed with RA or JIA. This difference is probably caused by a selection bias, as only patients who had underwent NCM were selected for this study. RA and JIA rarely present with RP as its first symptom. For this reason, NCM is usually not performed at first clinical evaluation [31]. When RP develops later during the disease process, NCM is not required to differentiate between PRP and SRP, as it is has already been established that a disease is present. Therefore RA and JIA patients may not have been included in this study.

The present study showed that ANA positivity was highly predictive of SRP. Anti-ENA antibodies were also strongly associated with SRP. These associations underline the importance of serology in children with RP. However, for most CTDs ANA or anti-ENA positivity is part of the diagnostic criteria [32]. Therefore, the involvement of ANA positivity in the diagnostics of CTDs has inevitably influenced the predictive value of ANA positivity for SRP. For this reason, this result should be interpreted with extreme caution.

Capillary loss on NCM was associated with SRP. However, capillary loss did not add to the predictive value of ANAs as shown by the multivariate analysis including ANA positivity. Other NCM parameters were not associated with SRP. It is plausible that the predictive value of ANA positivity was so strong, as it occurs so frequently in childhood rheumatic diseases, that it prevented other variables to be selected as independent predictors in this multivariate model. The multivariate analysis not including ANA positivity shows that this is likely true. In the multivariate analysis without ANA positivity, capillary loss was shown to be an independent predictor of SRP. Noteworthy for the models with and without ANA positivity is that they had high percentages of correctly predicted PRP (92% and 98% respectively), but quite low percentages of correctly predicted SRP (38% and 28% respectively). Because the multivariate model was likely influenced by the strong predictive value of ANA, an ROC analysis was also performed. An ROC analysis cannot have been influenced by ANA positivity. ROC and AUC analysis showed that capillary loss was an insufficient predictor of SRP.

The NCM finding capillary loss as a predictor for SRP had a high specificity and negative predictive value (NPV). However, the sensitivity and positive predictive value (PPV) were low. ANA screening performed better as a predictor for SRP with a higher sensitivity, specificity PPV and NPV. ANA screening and capillary loss combined as a predictor of SRP performed equally to ANA screening only as a predictor. The variations of NCM findings at different ages should be considered when interpreting this result [33, 34]. Specifically, Piotto et al. showed that age is positively correlated with the amount of capillaries in healthy children [33]. We were unable to analyze subgroups due to our small study population. In our study, the mean age of the SRP patients was lower than that of the PRP patients. This may have influenced our results. Furthermore, although the SRP patients had significantly fewer capillaries, they had a mean number of capillaries of 6.4 per mm. This is above the definition of capillary loss of < 6 per mm. It should also be noted that the ROC curve for the number of capillaries had an AUC of 0.65 that was not significant (*p* = 0.06). This shows that capillary loss has no value in distinguishing between PRP and SRP and this should be kept in mind when interpreting the sensitivity and specificity. Our study did show a trend towards a high specificity and NPV, which suggests NCM shows potential as a first screening method for SRP. However, because of the aforementioned points, definite conclusions about this cannot be made. A larger prospective study is needed to analyze whether NCM is a predictor of SRP.

Whereas studies in adults clearly show a relationship between giant capillaries on NCM and SRP [27], the present study found no evidence of this relationship in children. A possible explanation for this observation is that giant capillaries are not an early feature of the CTDs that children with RP are mostly at risk for. They are, however, an early feature of SSc, the most common CTD in adults with SRP [29]. Giant capillaries might still be helpful in defining a small subgroup of children with RP at risk of developing SSc. In our study, the one SSc patient did have giant capillaries, as well as three out of four MCTD patients and one of the two DM patients. This is in line with previous studies, which showed that a SSc pattern is common in MCTD and DM [27, 35–37]. It is also possible that although giant capillaries do not differentiate between PRP and SRP, they could still be predictive of SRP or a specific CTD. This can be seen in a recent study by Schonenberg-Meinema et al., which investigated whether NCM findings are abnormal in childhood-onset SLE and found that children with SLE had significantly more giant capillaries, abnormal capillaries and hemorrhages in comparison to healthy controls [38]. Although children with SLE had significantly more giant capillaries than healthy controls, the study did not find a significant correlation of RP and giant capillaries in the SLE patients [38]. The patient numbers of this study are too small to test for a relationship with individual CTDs, but studies with more participants and larger diagnostic subgroups might be capable of detecting such a relationship. More research is needed to determine whether giant capillaries are predictive of specific CTDs such as SSc.

It was established that NCM findings are poor predictors of SRP in children. However, SRP can be due to a large variety of CTDs. Juvenile SSc is rare, with an incidence of 0.27 per million per year [39]. In comparison, the incidence of childhood SLE is 0.3–0.9 per 100.000 children per year [40], the incidence of childhood MCTD is 0.1 per million children per year [41] and the incidence of childhood DM is 2–4 per million children per year [42]. It is possible that NCM is not as good a predictor in children as it is in adults, because children mostly develop CTDs that are less strongly associated with capillaroscopic abnormalities. Furthermore, our study only looked at SRP, meaning it analyzed the predictive value for all CTDs together. NCM could still be predictive of some individual CTDs. The current study was unable to analyze this hypothesis, because of the small patient group with SRP at follow up (*n* = 18). It might be of interest for future research to analyze the predictive value of NCM for each CTD individually when a larger study population of children with RP is available. The aforementioned study by Schonenberg-Meinema et al. investigated NCM findings in childhood-onset SLE in comparison to healthy controls [38]. Our study adds to theirs, because we compared to PRP patients, whereas their study compared to healthy controls. The study by Schonenberg-Meinema et al. emphasizes the importance of looking at specific NCM abnormalities. Because of the findings of this study, we believe NCM might be predictive of SLE in children with RP. The results of this study emphasize that it is worthwhile to analyze the predictive value of NCM for each CTD individually.

A limitation of this study is that the study population might be skewed towards SRP. All patients were referred to a tertiary center because they were suspected of having SRP. This may have led to overrepresentation of SRP patients, limiting the external validity for first line RP patients. Our study was also limited due to the retrospective design. Patient characteristics such as smoker status and family history of RP were not documented in the electronic patient files at the time of NCM and therefore unavailable to us. Another limitation of this study was that not all NCM were assessed using the same method. For the NCM performed until August 2013, dilated and giant capillaries were judged visually, whereas for the NCM performed after August 2013 they were measured apically. We expect that there are little differences between the visual judgement and measurements of giant capillaries. For dilated capillaries, however, there could be differences as these are visually less prominent. We found no significant association between SRP and dilated and giant capillaries. Therefore this limitation is not relevant for the outcome of our study. The number of capillaries was assessed over a 3 mm grid in both methods. Therefore, the use of different methods is highly unlikely to have affected the significant association of capillary loss with SRP. Lastly, the amount of parameters assessed with NCM was limited. Not all parameters defined by Piotto et al. were assessed during NCM [33]. More research should be performed to determine the value of NCM as a screening method when using the parameters defined by Piotto et al. [33].

## Conclusion

The aim of this study was to evaluate the predictive value of NCM in addition to ANAs positivity in differentiating between PRP and SRP. This study showed that NCM findings are insufficient predictors of SRP in children with RP and do not add to the predictive value of ANA positivity. However, NCM could be useful as a first screening method for SRP, due to its high specificity of 84%. Specific NCM findings might still be predictive of some individual CTDs, and future research should focus on this once a larger patient group is available. Whereas RP in adults is mostly associated with SSc, children with RP seem to mostly develop various other CTDs, of which MCTD, UCTD and SLE occur most frequently. In conclusion, NCM has potential as a screening method for SRP, but more research with a larger study population is required before we can conclude whether NCM can be used as a screening method.

## Data Availability

The dataset used and analyzed in the current study is available from the corresponding author on reasonable request.

## Abbreviations

ANAs: Antinuclear antibodies
CSQ: Connective tissue disease screening questionnaire
CTD: Connective tissue disease
DM: Dermatomyositis
ENAs: Extractable nuclear antigens
EPD: Electronic patient record
iSLE: Incomplete systemic lupus erythematosus
iSS: Incomplete Sjögren’s Syndrome
iSSc: Incomplete systemic sclerosis
JIA: Juvenile idiopathic arthritis
MCTD: Mixed connective tissue disease
NCM: Nailfold capillary microscopy
PM: Polymyositis
PRP: Primary Raynaud’s phenomenon
RA: Rheumatoid arthritis
RCS: Raynaud Condition Score
RP: Raynaud’s phenomenon
SLE: Systemic lupus erythematosus
SRP: Secondary Raynaud’s phenomenon
SS: Sjögren’s Syndrome
SSc: Systemic sclerosis
UCTD: Undifferentiated connective tissue disease
